# Neuropsychological outcomes from constant current deep brain stimulation for Parkinson's disease

**DOI:** 10.1002/mds.26827

**Published:** 2016-10-18

**Authors:** Alexander I. Tröster, Joseph Jankovic, Michele Tagliati, DeLea Peichel, Michael S. Okun

**Affiliations:** ^1^ Department of Clinical Neuropsychology and Barrow Center for NeuromodulationBarrow Neurological InstitutePhoenixArizonaUSA; ^2^ Baylor College of Medicine, Department of NeurologyHoustonTexasUSA; ^3^ Cedar‐Sinai Medical Center, Department of NeurologyLos AngelesCaliforniaUSA; ^4^ St. Jude Medical, Clinical StudiesPlanoTexasUSA; ^5^ Department of Neurology, Center for Movement Disorders and NeurorestorationUniversity of Florida SchoolGainesvilleFloridaUSA

**Keywords:** deep brain stimulation, parkinson's disease, neuropsychological outcomes, somatosensory temporal discrimination

## Abstract

**Objective:**

The aim of this study was to evaluate the neurobehavioral safety of constant‐current subthalamic deep brain stimulation and to compare the neuropsychological effects of stimulation versus electrode placement alone.

**Methods:**

A total of 136 patients with Parkinson's disease underwent bilateral subthalamic device implantation in this randomized trial. Patients received stimulation either immediately after device implantation (n = 101; active stimulation) or beginning 3 months after surgery (n = 35; delayed activation control). Patients were administered neuropsychological tests before, 3, and 12 months after device implantation.

**Results:**

Neuropsychological change in stimulation and control groups were comparable. Within‐group analyses revealed declines in category and switching verbal fluency in both groups, but only the stimulation group had letter verbal fluency and Stroop task declines. Depression symptom improvements occurred in both groups, but more often in the stimulation group. Letter fluency declines were associated with worse Parkinson's Disease Questionnaire Communication subscale scores. Baseline and 12‐month comparisons (in the combined group) revealed gains in verbal and visual delayed recall scores and improvement in depression symptoms, but decrements in verbal fluency and Stroop scores.

**Conclusions:**

Constant‐current bilateral subthalamic stimulation had a good cognitive safety profile except for decrements in verbal fluency and on the Stroop task. These abnormalities are related to device implantation, but stimulation likely had an additive effect. One year after surgery, the cognitive changes did not exert a detrimental effect on quality of life, although letter fluency declines were associated with communication dissatisfaction at 12 months. Improvement in depressive symptom severity appears dependent on stimulation and not placebo or lesion effects. © 2016 The Authors. Movement Disorders published by Wiley Periodicals, Inc. on behalf of International Parkinson and Movement Disorder Society.

Several randomized trials have shown voltage‐controlled subthalamic deep brain stimulation (STN DBS) devices to be superior to best medical therapy (BMT) in alleviating the motor symptoms of Parkinson's disease (PD) and in addressing levodopa‐related motor therapy complications (eg, dyskinesias) as well as in improving health‐related quality of life (QOL).^1^, ^2^, ^3^ Significant gains in QOL have been observed after DBS in comparison to BMT even in younger persons with PD who have previously experienced motor complications.^4^ A recent trial showed that constant current STN stimulation also improved motor function and QOL.^5^

Despite its positive impact on QOL and motor control, STN DBS has potential side effects, for example, mild, circumscribed, and often transient declines in cognition.^2^, ^3^, ^6^, ^7^, ^8^, ^9^, ^10^ Although memory, attention, and executive functions can be impacted by STN DBS, the most consistent (albeit not universal) declines involve verbal fluency.^11^, ^12^, ^13^, ^14^ Such declines occur in 25% to 50% of patients,^15^, ^16^ can persist for 3 to 5 years,^16^, ^17^ and might even worsen between 5 and 8 years after surgery.^18^

The neuropsychological effects of constant current DBS have not been documented. This study supplements the original study report^5^ by providing detailed neuropsychological outcome data. The study design facilitates the evaluation of competing explanations for verbal fluency decrements after STN DBS, for example, that verbal fluency decrements might represent a microlesion or implantation effect rather than a stimulation effect^19^ even though high but not low frequency stimulation may be associated with fluency decrements.^20^ The study also tested the hypothesis that verbal fluency decrements are a function of reduction in dopaminergic medication after DBS.^21^ The current study was also designed to document whether verbal fluency changes impact QOL. Finally, we address the proposal that overall cognitive outcome might be predicted by a combination of attention test findings, age, and dopaminergic medication response prior to surgery.^10^

## Patients and Methods

### Study Design

Study design and methodology have been previously published.^5^ Briefly, patients were randomly assigned in a 3:1 ratio to receive stimulation either immediately (7 days) after device implantation was completed (active stimulation; AS) or 3 months following surgery (delayed activation control; DA). Neuropsychological evaluations were completed at baseline, 3 months after surgery (comparing cognitive changes in AS and DA relative to baseline), and again 12 months after surgery (comparing cognitive change in the combined stimulation [Stim] group compared to baseline).

### Neuropsychological Evaluation

Baseline neuropsychological evaluation occurred 1 to 4 weeks prior to surgery. These data served not only as comparison points for the 2 postsurgical evaluations (day 90 and day 365) but also afforded a rigorous confirmation method that patients did not have significant cognitive impairment or untreated depression. It is emphasized that neuropsychological evaluation results at 90 days compared changes between the AS and DA groups relative to baseline, whereas evaluation at 365 days compared the entire group's performance to baseline. Thus, in the 365 day comparison, the majority of patients (the original AS group) had undergone 12 months of stimulation, but a minority (the original DA group) had undergone 9 months of stimulation. When alternate test forms were available, the order in which the forms were administered was randomized. In the case where only 2 alternate forms were available, the baseline and 12‐month evaluations used the same test form. It is noted that the use of alternate test forms minimizes test–retest practice effects, but does not completely eliminate factors such as “test wisdom.” Nonetheless, it is unlikely that test–retest influences would mask (or compensate) for cognitive declines when alternate forms are used. Similarly, it is improbable that disease progression would account for declines over 3‐ and 9‐month test–retest intervals given the typically limited change in patients during such an interval.^22^

The neuropsychological tests selected evaluated key domains of cognition, including overall level of cognitive function (Dementia Rating Scale–2nd edition [DRS‐2]^23^, attention and working memory (Stroop Color and Word Test 24 and Trailmaking Test^25^, executive functions (Delis‐Kaplan Executive Function System),^26^ verbal fluency tests (Wisconsin Card Sorting Test–64 card version^27^ and Frontal Systems Behavior Scale^28^, episodic verbal and visual memory (Wechsler Memory Scale, Third Edition, Abbreviated [WMS‐III‐A]^29^ and Hopkins Verbal Learning Test–Revised [HVLT‐R]^30^, depression (Hamilton Depression Inventory [HDI]^31^, and measures of intelligence (Wechsler Abbreviated Scale of Intelligence [WASI]^32^. WASI and visual confrontation naming (Boston Naming Test)^33^ were administered at baseline to characterize the sample and to facilitate dementia screening. Tests were administered in a standard order across all sites.

### Statistical Analyses

Sample size was determined so as to allow detection of a 3‐hour difference in on‐time without dyskinesias between baseline and follow‐up with a power of 80% at a statistical significance level of .05, and assuming a 15% dropout rate. Analyses were conducted using the standardized test scores traditionally used in clinical practice. Such scaled scores, *T* scores, and index scores, depending on the test, are corrected for demographic factors such as age, education, and/or gender. The primary neuropsychological analyses comparing change from baseline to 90 days between the AS and DA groups were achieved via analyses of covariance using baseline score and study site as covariates. Because change score analyses between groups do not fully address safety information, these primary analyses were supplemented by within‐group *t* tests (to determine whether each group's scores had changed significantly from baseline) and by chi‐square analyses that compared the frequency of changes (no change, decline by 1 standard deviation [SD] or more, improvement by 1 SD or more) in the 2 groups. Changes of 1 SD are typically considered in neuropsychology to be of possible clinical significance. After 90 days, the DA group received active stimulation (after the 3‐month neuropsychological evaluation) and were evaluated in combination with the AS group at 12 months.

To determine whether verbal fluency declines influenced satisfaction with communication (per report on the Parkinson's Disease Questionnaire [PDQ‐39]), independent *t* tests were performed at 12 months to compare mean change in PDQ Communication between verbal fluency decliners and nondecliners. Pearson correlations were used to determine whether statistically significant associations existed between verbal fluency and changes in depression, attention, and levodopa‐equivalent dosage. To address whether, as shown in a prior study,^10^ changes in cognition might be predicted by baseline age, levodopa response, and attention, regression analyses were used with age, baseline levodopa response, and attention composite scores as predictors. Outcome (cognition) was defined in 2 ways. The first was a clinically practical and convenient measure that was the change on a cognitive screening examination (DRS‐2). The second definition of cognitive outcome was based on a more rigorous neuropsychological test‐based composite based on change in executive function and memory standardized scores: sum of the standardized Wisconsin Card Sorting Test perseverative errors, letter fluency, semantic fluency, HVLT‐R total immediate and delayed recall, WMS Logical Memory and Family Pictures immediate and delayed recall scores at 3 months minus the same scores obtained at baseline (note that attention measures were not included so as not to confound outcome and predictors). The predictors were age, baseline percent levodopa response, and a composite of attention and working memory (mean of the standardized Stroop interference and Trailmaking test part B scores).

## Results

The demographic and disease characteristics at baseline (see Table 1) did not differ significantly between the AS and DA groups.

**Table 1:** Baseline demographic and disease characteristics of the stimulation and control groups

	Stimulation, n = 101	Control, n = 35	*P* value
Gender			
Male, n (%)	63 (62.4)	21 (60.0)	.803
Female, n (%)	38 (37.6)	14 (40.0)	
Race			
White, n (%)	91 (90.1)	31 (88.6)	.755^a^
African American, n (%)	1 (1.0)	0 (0)	
Hispanic, n (%)	8 (7.9)	3 (8.6)	
Other, n (%)	1 (1.0)	1 (2.9)	
Age, y			
Mean (SD)	60.6 (8.3)	59.5 (8.2)	.519
Range	41‐78	41‐76	
Years since symptom onset			
Mean (SD)	12.1 (4.9)	11.7 (4.1)	.684
Range	5‐29	5‐19	

a White versus nonwhite.

**Figure 1:**
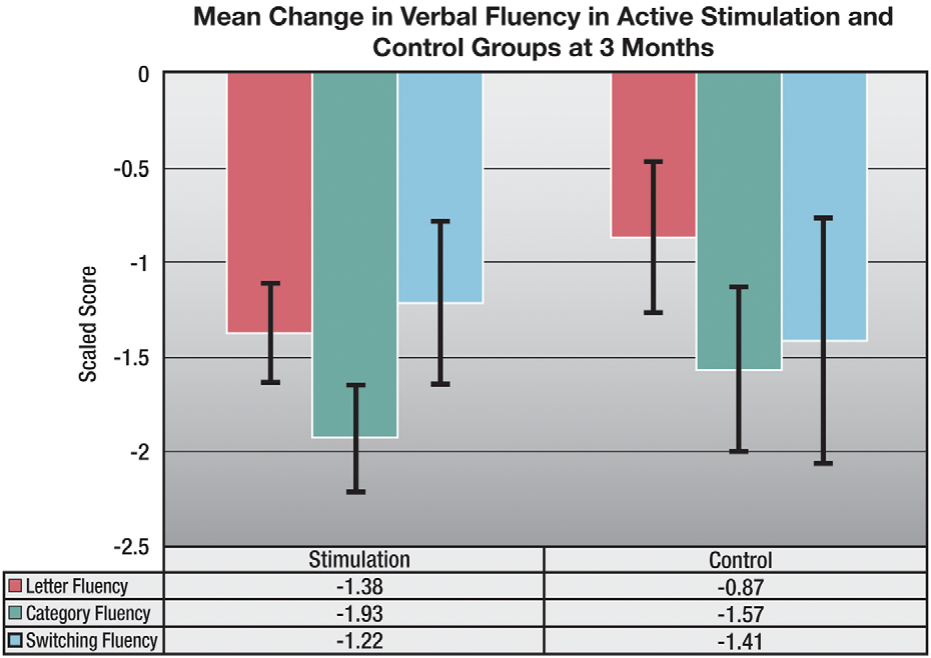
Mean change in verbal fluency in active stimulation and control groups at 3 months.

Levodopa‐equivalent dosage was significantly reduced from baseline in both groups, but the reduction was significantly greater in the stimulation versus the control group (*P* < .0001; see Supplemental Table 1). As shown in Table 2, there were no significant differences between the stimulation and control groups' baseline neuropsychological test scores. Analyses of covariance on neuropsychological change scores at 3 months did not yield significant interaction terms, but depression symptom severity as measured with the HDI improved significantly only in the stimulation group, yielding a significant group x time interaction (*P* = .005; see Table 2). Although the interaction term was not significant, within‐group *t* tests revealed that both the stimulation and control groups demonstrated significant declines in category and switching verbal fluency (see Figure 1). The stimulation, but not the control group, showed evidence of significant declines on all parts of the Stroop task and on the letter verbal fluency task. Also unique to the stimulation group were statistically significant increases in the scores for delayed story recall (Logical Memory II) and immediate and delayed recall of pictured scenes (Family Pictures I and II). The control group, but not the stimulation groups, experienced a decline in the DRS initiation/perseveration score.

**Table 2:** Neuropsychological performance at baseline and 3 months in the stimulation and control groups and 12 months, mean (SD)

	Stimulation		Control		12 months	
	Baseline	3 months	Baseline	3 months	Baseline	Follow‐Up
Dementia Rating Scale scaled scores						
Attention	10.9 (2.2)	10.9 (2.1)	10.7 (2.5)	10.9 (2.1)	11.0 (2.0)	11.0 (2.4)
Initiation	9.5 (2.3)	9.1 (2.8)	9.8 (2.3)	8.5 (2.9)^a^	9.6 (2.3)	9.0 (2.8)^a^
Construction	9.3 (1.7)	9.4 (1.4)	9.4 (2.0)	9.2 (2.0)	9.4 (1.6)	9.3 (1.7)
Conceptualization	9.2 (2.2)	9.1 (2.0)	9.1 (2.5)	9.2 (2.2)	9.2 (2.1)	9.6 (2.3)
Memory	9.1 (3.0)	9.4 (3.2)	8.5 (3.1)	9.3 (2.9)	9.1 (2.9)	9.4 (2.9)
Total	9.4 (2.8)	9.5 (5.6)	9.4 (2.6)	9.2 (3.6)	9.5 (3.2)	9.4 (3.5)
Stroop *T* scores						
Word	38.8 (11.3)	37.4 (10.5)	39.3 (10.1)	39.0 (10.4)	39.1 (10.8)	35.3 (11.8)^a^
Color	39.5 (10.3)	37.4 (10.7)^a^	39.0 (9.8)	37.7 (10.6)	39.4 (10.2)	35.3 (11.0)^a^
Color‐Word	44.4 (9.4)	41.5 (9.1)^a^	44.8 (8.9)	42.4 (10.1)	44.7 (9.2)	41.6 (10.2)^a^
Interference	47.7 (6.9)	45.9 (7.9)^a^	47.0 (8.4)	46.6 (8.2)	47.9 (7.0)	46.9 (8.3)
Delis‐Kaplan Executive Function System scaled scores						
Letter fluency	10.6 (4.2)	9.1 (3.7)^a^	10.5 (4.2)	9.6 (4.5)	10.5 (4.2)	9.1 (3.9)^a^
Category fluency	10.6 (3.8)	8.7 (3.6)^a^	10.2 (3.3)	8.8 (3.4)^a^	10.5 (3.7)	8.6 (3.6)^a^
Switching fluency	10.4 (3.9)	9.2 (4.1)^a^	11.4 (2.5)	9.5 (3.5)^a^	10.7 (3.5)	9.0 (3.9)^a^
Switching accuracy	10.2 (3.6)	9.5 (3.9)	11.1 (2.8)	9.5 (3.3)^a^	10.5 (3.4)	9.2 (3.9)^a^
Wisconsin Card Sorting Test						
Categories (of 6)	2.71 (1.50)	2.54 (1.58)	3.13 (1.41)	3.13 (1.45)	2.82 (1.49)	2.64 (1.7)
Perservative (errors)						
Raw scores	11.1 (7.4)	10.4 (6.5)	10.5 (6.6)	9.2 (6.0)	11.1 (7.5)	10.7 (6.3)
*T* scores	46.5 (13.8)	47.5 (13.2)	46.1 (11.4)	49.5 (12.6)	46.1 (12.9)	48.1 (12.8)
Nonperservative (errors)						
Raw scores	9.1 (5.8)	10.2 (6.1)	7.8 (5.3)	8.7 (5.0)	8.8 (5.8)	9.8 (6.0)
*T* scores	45.5 (13.3)	42.9 (13.2)	47.9 (10.6)	44.8 (9.7)	46.0 (12.4)	44.4 (12.2)
Trailmaking test *T* scores						
Trailmaking A	44.6 (11.6)	43.1 (12.4)	41.5 (12.7)	40.9 (11.3)	43.8 (12.0)	42.7 (12.9)
Trailmaking B	41.6 (12.6)	40.7 (14.3)	39.2 (12.4)	36.7 (15.7)	41.6 (11.8)	40.3 (14.0)
Hopkins Verbal Learning Test–Revised *T* scores						
Total recall	39.1 (11.5)	40.0 (11.3)	36.8 (11.0)	38.9 (10.2)	38.5 (11.3)	39.5 (11.3)
Delayed recall	40.5 (12.8)	39.3 (13.0)	38.9 (11.0)	39.3 (11.2)	40.6 (12.3)	40.4 (13.0)
Retention	44.7 (14.3)	42.6 (13.2)	42.2 (10.9)	44.6 (12.1)	44.9 (13.4)	44.6 (13.3)
Recognition	41.2 (12.4)	42.5 (11.6)	43.2 (13.3)	45.3 (13.3)	41.7 (12.6)	43.1 (12.6)
Wechsler Memory Scale (III‐A) scaled scores						
Logical Memory I	9.7 (3.7)	9.9 (3.6)	10.2 (2.7)	10.1 (2.7)	9.9 (3.4)	10.5 (3.2)^a^
Logical Memory II	10.3 (3.4)	10.9 (3.4)^a^	10.7 (2.9)	10.9 (3.1)	8.8 (3.3)	9.5 (3.5)^a^
Family Pictures I	8.9 (3.6)	9.6 (3.2)^a^	8.8 (2.2)	8.8 (2.8)	10.6 (3.2)	11.2 (3.3)^a^
Family Pictures II	9.0 (3.4)	9.8 (3.3)^a^	8.8 (2.7)	9.2 (3.2)	8.9 (3.3)	9.7 (3.5)^a^
Hamilton Depression Inventory *T* score						
Total*^a^	66.1 (13.2)	57.4 (13.7)^a^	69.3 (13.7)	66.2 (11.9)	66.9 (13.3)	60.2 (14.5)^a^
Frontal Systems Behavior Scale *T* scores						
Apathy	64.8 (18.3)	61.3 (16.1)^a^	69.0 (16.8)	65.8 (14.2)	65.5 (17.5)	64.8 (16.2)
Disinhibition	56.6 (18.3)	55.6 (15.2)	60.4 (13.4)	60.3 (14.7)	58.1 (17.4)	58.1 (16.9)
Executive dysfunction	62.4 (16.0)	59.7 (14.1)	64.4 (17.6)	65.4 (13.3)	62.7 (16.0)	61.3 (15.0)
Total	64.4 (18.2)	61.2 (15.8)	68.3 (14.8)	66.4 (13.6)	65.2 (17.1)	63.6 (16.8)
Wechsler Abbreviated Scale of Intelligence *T* scores						
IQ	–	–	–	–	104.9 (17.4)	103.7 (16.9)
Vocabulary	–	–	–	–	53.8 (10.1)	51.0 (10.8)^a^
Matrix reasoning	–	–	–	–	51.6 (12.0)	52.0 (11.0)

IQ, intelligence quotient.

a *P* < .05 for paired *t* tests addressing change within groups.

**P < .05 for analysis of covariance for group by time, adjusting for site and baseline score.

**Figure 2:**
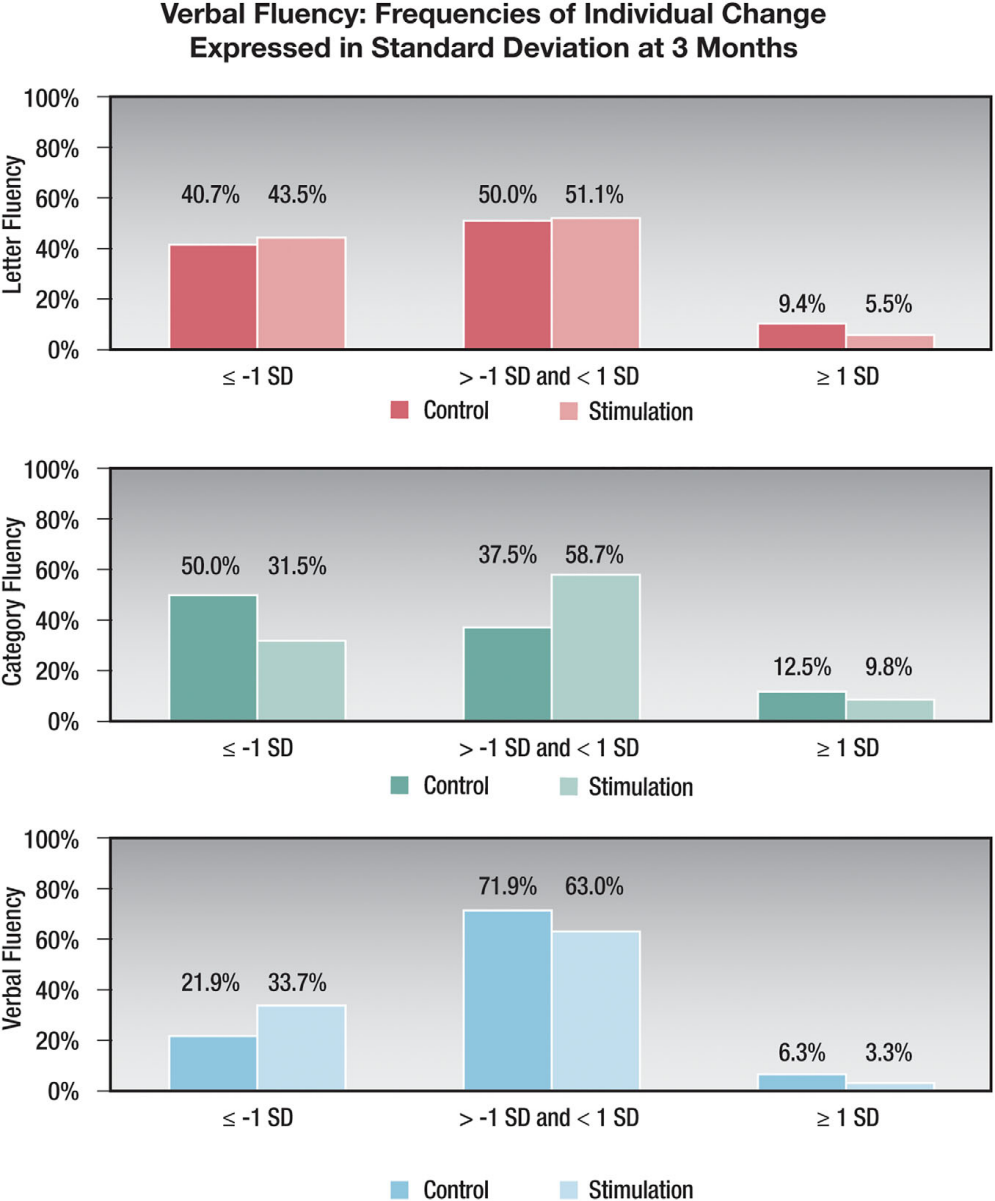
Verbal fluency: frequencies of individual change expressed in standard deviations at 3 months. SD, standard deviation.

The magnitude of the verbal fluency changes was of likely clinical significance ( > 1 SD decline) in 16% to 40% of the patients, depending on the task (see Supplemental Table 2 and Fig. 2). In contrast, the Stroop interference score declined in only about 9% of patients in both groups. Mean improvements in the WMS memory scores, despite their statistical significance, were unlikely of clinical significance because 0% of the changes exceeded 1 SD in either direction. Depression score changes of clinical significance ( > 1 SD improvement) were observed in 30% of the control group and 43% of the stimulation group.

Changes in letter fluency score from baseline to 3 months (in the entire study sample) were not significantly correlated with age or changes on tests of complex attention/working memory (Trailmaking part B, Stroop Interference; all *r* < 0.1, *P* > .42). Similarly, category fluency change at 3 months was not significantly correlated with either age or attention/working memory test score changes (all *r* < 0.17, *P* > .08), although the association between change in Trailmaking part B and category fluency approached significance (*r* = 0.16, *P* = .08). Neither letter (*r* = 0.02, *P* = .85) nor category fluency changes (*r* = −0.01, *P* = .92) were significantly associated with quality of life (PDQ‐39 Communication) change. There was only a trend for a negative association between switching fluency declines and changes in PDQ‐39 Communication scores (*r* = −0.22, *P* = .08). Fluency changes were not associated with changes in the medication expressed in levodopa‐equivalent units (all *r* < 0.06, *P* > .52; see Supplemental Table 3).

### Baseline Predictors of Cognitive Change at 90 Days

The change in overall level of cognitive functioning (DRS‐2 total score) was not significantly associated with a combination of baseline levodopa response, age, and an average composite of attention/working memory. A composite of average change on executive and memory tests similarly was not significantly associated with the predictors of levodopa response, age, and attention/working memory. Overall change in cognition (DRS‐2) was not associated with change in depression score (*r* = 0.002; *P* > .98).

### Neuropsychological Changes From Baseline to 12 Months

**Figure 3:**
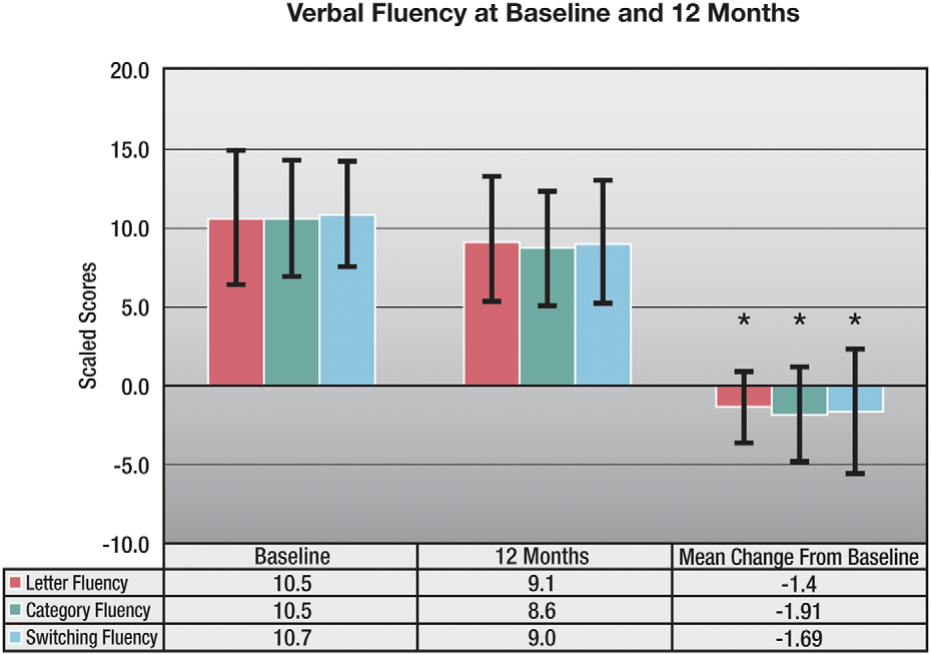
Verbal fluency at baseline and 12 months.

Baseline and 12‐month scores are shown in Table 2. The WASI Vocabulary *T* score declined significantly for the entire sample from baseline (*P* = .004). On the Stroop task, there were significant declines in the word, color, and color‐word (all *P* < .001) portions, but not on the interference part of the task (*P* = .26). Declines in excess of 1 SD occurred in about 18% of the patients on each of the first 3 parts of the Stroop test (see Supplemental Table 2). The Hamilton Depression Inventory score was significantly lower (improved) at 12 months than at baseline (*P* < .001), with improvements of 1 SD or more seen in 39% of patients. The 4 WMS‐III‐A subtest scores all increased from baseline (all *P* < .01), but individually, 0% of patients showed changes exceeding 1 SD. All 3 verbal fluency tasks revealed significant declines at 12 months when compared with baseline (all *P* < .001; see Fig. 3). Declines greater than 1 SD occurred in 31% of patients in letter fluency, 36% in category fluency, and 43% in switching fluency (see Supplemental Fig. 1). For the groups showing greater than 1 SD loss versus less than 1 SD loss in verbal fluency scores, only those showing declines greater than 1 SD in letter fluency showed a statistically significance change (greater dissatisfaction) on the PDQ‐39 Communication scale (*P* < .026) at 12 months (see Supplemental Table 4).

## Discussion

The key findings of this study were consistent with the prior literature suggesting that STN DBS is relatively safe from a cognitive standpoint.^4^, ^7^, ^8^, ^34^ The majority of cognitive test scores did not reveal significant changes at 3 or 12 months after surgery. There were, however, declines on tests of verbal fluency, processing speed, and attention/working memory. This study adds important information to the existing literature because the delayed stimulation control design of this trial revealed that when changes occurred in cognition, they appeared in both the active stimulation and control groups. Thus, a “microlesion effect” or some other aspect of electrode implantation might underlie some cognitive changes as has been suggested previously.^5^, ^19^ Importantly, however, such microlesion surgical effects do not completely explain cognitive decline because the letter fluency and Stroop task declines occurred only in the stimulation group. The occurrence of a significant decline in letter fluency in the stimulation but not the control group is unlikely to reflect the larger sample size (and greater statistical power) in the stimulation than control group (patients were randomized in a 3:1 ratio to these groups). Although the effect size of the decline was moderate (d = 0.38) in the stimulation group, it was small in the control group (d = 0.21). Consequently, although proportions of persons showing less than 1 SD and greater then 1 SD changes in letter fluency were similar in the 2 groups, the decline was larger in the stimulation than in the control group. Similarly, although both effect sizes were small, the decline in the Stroop interference task was greater in the stimulation (d = 0.24) than in the control group (d = 0.05).

Consistent with prior studies,^11^, ^13^ verbal fluency changes of possible clinical significance were common, occurring in 16% to 40% of patients, depending on the verbal fluency task. The exact mechanisms underlying the verbal fluency changes could not be ascertained in this study, but given the changes on the Stroop tasks, factors such as executive function and processing speed likely play a role in these changes. Although a recent study failed to find associations between verbal fluency changes and a few executive function measures,^35^ such a proposal is consistent with the notion that fluency tasks, although weakly to moderately associated with executive function measures, address a unique aspect of cognition^36^ and consistent with the finding that the executive process of switching between word subcategories during verbal fluency tasks is disrupted following STN DBS.^37^ The durability of the verbal fluency decline after STN DBS over 12 months of follow‐up was consistent with prior work showing that declines may persist up to 5 years after surgery^17^ and that the difference in verbal fluency deficits between operated versus nonoperated patients may become more exaggerated over time.^18^

Of the significant neuropsychological test score changes, the majority involved declines. However, the 4 WMS‐III‐A subtest scores (immediate and delayed recall of pictured scenes and stories) improved in the stimulation group at 90 days. This change may represent practice effects because gains were not observed on other memory tasks (HVLT‐R) and it was not likely clinically meaningful with no patients experiencing gains exceeding 1 SD.

One goal of neuropsychological evaluation in DBS candidates is the accurate prediction of which individuals will experience marked cognitive changes during therapeutic stimulation. The metrics to predict these changes have has been poorly defined and have to date failed to provide reliable predictors of which individuals will experience marked cognitive changes (in the absence of operative or perioperative complications). Numerous risk factors have been proposed for cognitive decline, albeit inconsistently, including age older than 69 years,^38^, ^39^ preoperative cognitive deficit,^37^, ^40^ higher stimulation frequency,^41^ stimulation amplitude and pulse width,^42^ stimulation in the anteroventral STN,^43^ baseline levodopa dose,^39^ and axial symptom severity.^39^ Smeding and colleagues^10^ reported that baseline neuropsychological‐, patient‐, and disease‐related variables considered in tandem might better predict the risk of overall cognitive decline.^10^ Specifically, they reported that attention scores (Stroop and Trailmaking composite), age, and levodopa response best predicted the risk for multivariate‐defined cognitive decline.^10^ Unfortunately, our study, similar to that of Odekerken and colleagues,^12^ was not able to replicate this finding using either a cognitive screening measure or a test score composite as outcome. One possible reason for the failure to replicate the findings may be that cognitive outcome was defined differently in the 2 studies.

Depression symptoms, as measured by the HDI, improved only in the stimulation group at 90 days and remained significantly improved at 12 months in the entire sample. Consequently, it is likely that stimulation might exert a beneficial effect on these symptoms. Whether the effect is generated by directly altering activity in the mesolimbic circuits, or indirectly (because of greater motor symptom relief or levodopa reduction in the stimulated group) is unknown. It should be noted that a significant proportion of patients in the control group also reported improvements in depressive symptoms—in this case, it is even more difficult to disentangle direct, indirect, and potential placebo effects. Improvements on self‐report symptom scales after STN DBS have been previously reported,^34^, ^44^ but it is important that symptoms of depression are not equivalent to the syndrome of depression or to an index of caseness (ie, the number of persons having the condition of interest).^45^ Nonetheless, this study had a low incidence of depression ( < 1% for serious adverse events across 12 months; 4% in the stimulation group and 9% in the entire sample between 3 and 12 months for nonserious events^5^, indeed at most time points lower than the 7% to 8% figures reported in meta‐analyses.^46^, ^47^

In conclusion, STN DBS, using a constant current device, appears to be reasonably cognitively safe, but mild changes in verbal fluency were common and mild declines in processing speed were evidenced. These changes can be attributed to both electrode placement and stimulation. Slight improvements in memory scores were observed, however, they did not exceed those typically expected from practice effects. The incidence of depression up to 1 year after surgery was low, and stimulation improved depressive symptoms. Neurobehavioral changes did not resolve within 12 months following DBS surgery.

## Acknowledgment

The authors thank Michael Kutner, Ph.D. for assistance with statistical analyses.

## Authors Roles

1. Research Project: A. Conception, B. Organization, C. Execution; 2. Statistical Analysis: A. Design, B. Execution, C. Review and Critique; 3. Manuscript Preparation: A. Writing the First Draft, B. Review and Critique.

A.I.T.: 1A, 2A,C, 3A,B

J.J.: 1A, 2C, 3B

M.T.: 1A, 2C, 3B

D.P.: 1A,B,C, 2C, 3B

M.S.O.: 1A, 2C, 3B

## Full financial disclosure for the previous 12 months

Drs. A.I.T. and M.T. are paid consultants for St. Jude Medical. A.I.T. has served as a consultant and/or on the scientific advisory boards for Medtronic, Boston Scientific, Teva, Takeda, Theravance, and Pfizer. He has received grant/research support support from National Institutes of Health, the Michael J. Fox Foundation, the Barrow Neurological Foundation, Glaxo Smith Kline, and Medtronic. A.I.T. has received book royalties from Cambridge University Press and Oxford University Press. M.T. has received honoraria from Medtronic Inc. J.J. is a consultant for Allergan, Chelsea Therapeutics, EMD Serono, Lundbeck Inc., Merz Pharmaceuticals, and Teva Pharmaceutical Industries Ltd; has received grants from Allergan, Allon Therapeutics, Ceregene Inc., Chelsea Therapeutics, Diana Helis Henry Medical Research Foundation, EMD Serono, Huntington's Disease Society of America, Huntington Study Group, Impax Pharmaceuticals, Ipsen Limited, Lundbeck Inc., Michael J. Fox Foundation for Parkinson Research, Medtronic, Merz Pharmaceuticals, National Institutes of Health, National Parkinson Foundation, Neurogen, St. Jude Medical Neuromodulation Division, Teva Pharmaceutical Industries Ltd, University of Rochester, and the Parkinson Study Group; and has received royalties from Elsevier, Lippincott Williams and Wilkins, and UpToDate. M.S.O. serves as a consultant for the National Parkinson Foundation and has received research grants from National Institutes of Health, National Parkinson Foundation, the Michael J. Fox Foundation, the Parkinson Alliance, Smallwood Foundation, the Bachmann‐Strauss Foundation, the Tourette Syndrome Association, and the University of Florida Foundation. M.S.O. has previously received honoraria, but in the past >60 months has received no support from industry. M.S.O. has received royalties for publications with Demos, Manson, Amazon, Smashwords, Books4Patients, and Cambridge (movement disorders books). M.S.O. is an associate editor for the *New England Journal of Medicine Journal Watch Neurology*. M.S.O. has participated in Continuing Medical Education and educational activities on movement disorders (in the past 36) months sponsored by PeerView, Prime, Quantia, Henry Stewart, and by Vanderbilt University. The institution and not M.S.O. receives grants from Medtronic, Abbvie, and Adanced Neuromodulation Systems/St. Jude, and the PI has no financial interest in these grants. M.S.O. has participated as a site principal investigator and/or co‐investigator for several National Institutes of Health, foundation, and industry sponsored trials over the years but has not received honoraria. D.P. is an employee of St. Jude Medical Inc.

## Supporting information

Additional Supporting Information may be found in the online version of this article at the publisher's web‐site.

Supplementary Information Figure 1.

Supplementary Information Table 1.

Supplementary Information Table 2.

Supplementary Information Table 3.

Supplementary Information Table 4.
