# Supplementary figures and images for: Neuropsychological outcomes from constant current deep brain stimulation for Parkinson's disease
Source: Mov Disord. 2016 Oct 18;32(3):433–40. doi: 10.1002/mds.26827 (PMC5363377; doi:10.1002/mds.26827)

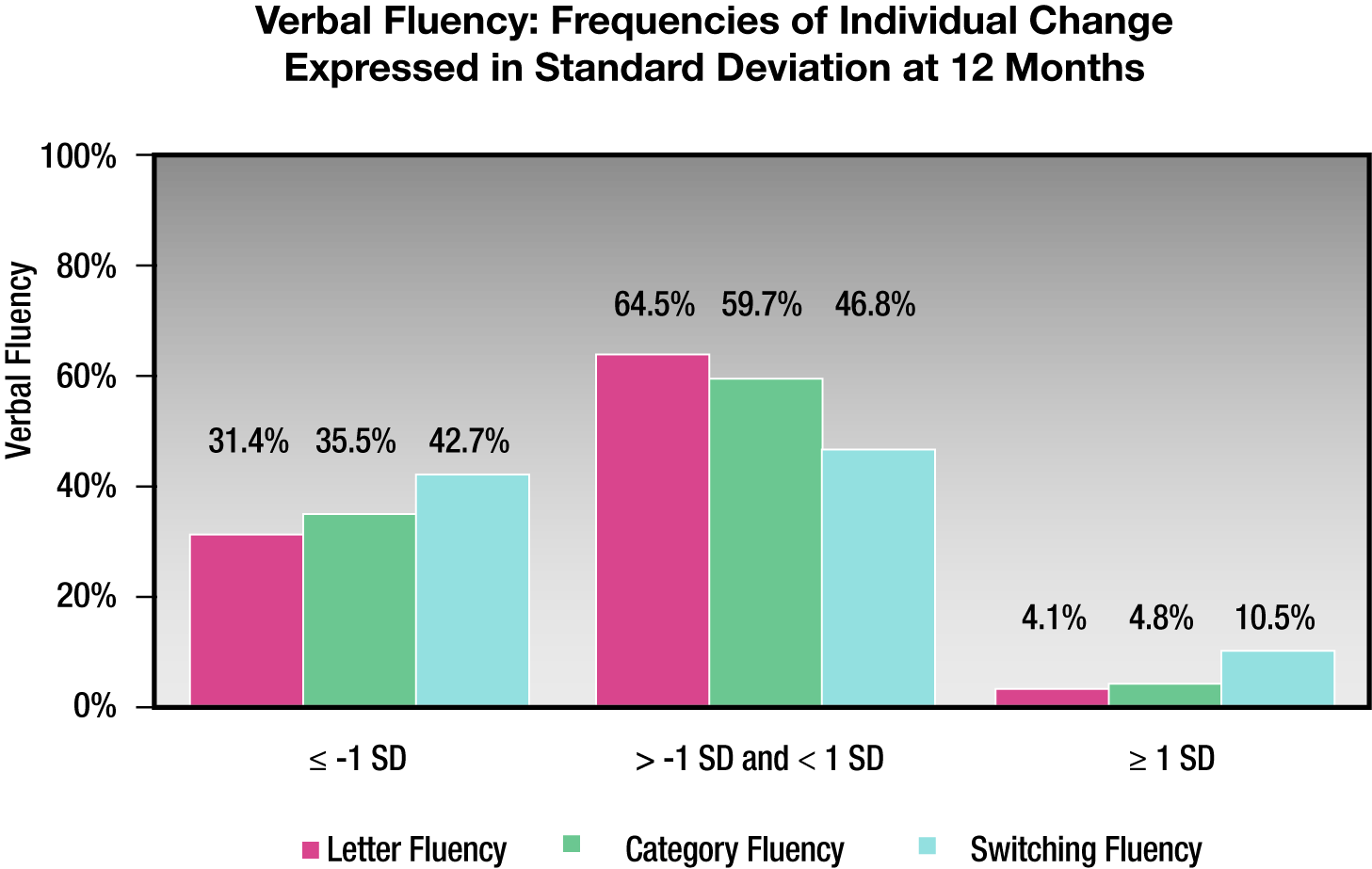

Supplement: Supplementary file 1 — Supplementary Information Figure 1. [file MDS-32-433-s001.tif]
