# Supplementary material for: Neuropsychological outcomes from constant current deep brain stimulation for Parkinson's disease
Source: Mov Disord. 2016 Oct 18;32(3):433–40. doi: 10.1002/mds.26827 (PMC5363377; doi:10.1002/mds.26827)
Supplement: Supplementary file 2 — Supplementary Information Table 1. [file MDS-32-433-s002.docx]

Table 1 Supplemental: Baseline and Change from Baseline in Total Daily Levodopa Dose Equivalent

| **Patients** | **Baseline** | **3 Months** | | **12 Months** | |
| --- | --- | --- | --- | --- | --- |
|  | | Actual | Change | Actual | Change |
| **Stim** | | | | | |
| N | 101 | 101 | 101 | 99 | 99 |
| Mean ± SD | 1311 ± 615 | 864 ± 551 | -446 ± 543* | 850 ± 509 | -474 ± 548* |
| **Control** | | | | | |
| N | 35 | 35 | 35 | 35 | 35 |
| Mean ± SD | 1459 ± 991 | 1272 ± 608 | -187 ± 850 | 881 ± 565 | -578 ± 959* |
| **All** | | | | | |
| N | 135* | na |  | 134 | 134 |
| Mean ± SD | 1354 ± 729 |  |  | 858 ± 522 | -501 ± 677* |

* Denotes a significant change from baseline (p<0.001).
