# Supplementary material for: Neuropsychological outcomes from constant current deep brain stimulation for Parkinson's disease
Source: Mov Disord. 2016 Oct 18;32(3):433–40. doi: 10.1002/mds.26827 (PMC5363377; doi:10.1002/mds.26827)
Supplement: Supplementary file 3 — Supplementary Information Table 2. [file MDS-32-433-s003.docx]

Table 2 Supplemental: Frequency and Proportions of Cognitive Test Score Changes at 3 and 12 Months [n (%)]

|  | **Stimulation (3-month)** | | | **Control (3-month)** | | | **Overall (12-month)** | | |
| --- | --- | --- | --- | --- | --- | --- | --- | --- | --- |
| Characteristic | Change from Pre-Op to 3 month | | | Change from Pre-Op to 3 month | | | Change from Pre-Op to 12 months | | |
|  | ≤ -1 SD | >-1 SD and <1SD | ≥ 1 SD | ≤ -1 SD | >-1 SD and <1SD | ≥ 1 SD | ≤ -1 SD | >-1 SD and <1SD | ≥ 1 SD |
| **Dementia Rating Scale**  **Scaled scores** |  |  |  |  |  |  |  |  |  |
| Attention | 11 (12.7) | 66 (75.9) | 10(11.5) | 3(9.7) | 26 (83.9) | 2(6.4) | 11(9.5) | 94 (81.0) | 11(9.5) |
| Initiation | 19 (21.9) | 55 (63.2) | 13(14.9) | 9(29.1) | 21 (67.7) | 1(3.2) | 21(18.1) | 87 (75.0) | 8(6.9) |
| Construction | 11 (12.6) | 64 (73.6) | 12(13.8) | 5(16.1) | 23 (74.2) | 3(9.7) | 15(13) | 88 (76.5) | 12(10.4) |
| Conceptualization | 12 (13.8) | 64 (73.6) | 11(12.6) | 6(19.4) | 19 (61.3) | 6(19.3) | 10(8.6) | 93 (80.2) | 13(11.2) |
| Memory | 19 (21.8) | 43 (49.4) | 25(28.7) | 7(22.6) | 12 (38.7) | 12(38.7) | 23(19.8) | 68 (58.6) | 25(21.5) |
| **Stroop T-Scores** |  |  |  |  |  |  |  |  |  |
| Word Score | 10 (10.8) | 81 (87.1) | 2(2.2) | 2(6) | 29 (87.9) | 2(6.1) | 22(17.8) | 98 (79.0) | 4(3.2) |
| Color Score | 15 (16.1) | 71 (76.3) | 7(7.5) | 2(6.1) | 31 (93.9) | 0(0) | 23(18.7) | 99 (80.5) | 1(0.8) |
| Color-Word Score | 15 (16.2) | 73 (78.5) | 5(5.4) | 6(18.2) | 24 (72.7) | 3(9.1) | 24(19.5) | 94 (76.4) | 5(4.1) |
| Interference | 11 (12) | 75 (81.5) | 6(6.5) | 3(9.1) | 27 (81.8) | 3(9.1) | 15(12.3) | 94 (77.1) | 13(10.6) |
| **Delis-Kaplan Executive Function Scale**  **Scaled scores** |  |  |  |  |  |  |  |  |  |
| Letter Fluency | 31 (33.7) | 58 (63.0) | 3(3.3) | 7(21.9) | 23 (71.9) | 2(6.3) | 39(31.4) | 80 (64.5) | 5(4) |
| Category Fluency | 40 (43.5) | 47 (51.1) | 5(5.4) | 13(40.7) | 16 (50.0) | 3(9.4) | 44(35.5) | 74 (59.7) | 6(4.8) |
| Switching Fluency Total Correct | 29 (31.5) | 54 (58.7) | 9(9.8) | 16(50.0) | 12 (37.5) | 4(12.5) | 53(42.7) | 58 (46.8) | 13(10.5) |
| Switching Accuracy | 28 (30.4) | 50 (54.4) | 14(15.2) | 14(45.2) | 13 (41.9) | 4(13) | 46(37.4) | 62 (50.4) | 15(12.2) |
| **Wisconsin Card Sorting Test (WCST-64)** |  |  |  |  |  |  |  |  |  |
| Categories Raw Scores | 16 (17.2) | 71 (76.3) | 6(6.5) | 6(18.8) | 22 (68.8) | 4(12.5) | 21 (16.8) | 92( 73.6) | 12 (9.6) |
| Perseverative errors raw score | 35 (37.6) | 29 (31.2) | 29(31.2) | 14(43.8) | 11 (34.4) | 7(21.9) | 47 (37.6) | 36 (28.8) | 42 (33.6) |
| Perseverative errors t score | 21 (22.6) | 50 (53.8) | 22(23.7) | 4(12.6) | 21 (65.6) | 7(21.9) | 19(15.5) | 78 (63.4) | 26(21.2) |
| Non-Perservative Raw Scores | 27 (29.1) | 26 (28.0) | 40(43) | 9(28.2) | 7 (21.9) | 16(50) | 42 (33.6) | 28 (22.4) | 55 (44.0) |
| Non-Perservative T-Scores | 22 (23.7) | 56 (60.2) | 15(16.2) | 9(28.2) | 21 (65.6) | 2(6.2) | 33(26.9) | 70 (56.9) | 20(16.2) |
| **Trailmaking Test T-Scores** |  |  |  |  |  |  |  |  |  |
| Trailmaking A | 15 (16.2) | 68 (73.1) | 10(10.8) | 6(18.8) | 22 (68.8) | 4(12.5) | 20(16.3) | 86 (69.9) | 17(13.8) |
| Trailmaking B | 15 (16.5) | 63 (69.2) | 13 (14.3) | 7(23.3) | 20 (66.7) | 3 (10.0) | 18 (15.4) | 82 (70.1) | 17 (14.5) |
| **Hopkins Verbal Learning Test –Revised**  **T-Scores** |  |  |  |  |  |  |  |  |  |
| Total Recall | 15 (15.8) | 62 (65.3) | 18(18.9) | 2(6.2) | 23 (71.9) | 7(21.9) | 22(17.5) | 78 (61.9) | 26(20.6) |
| Delayed Recall | 17 (17.9) | 63 (66.3) | 15(15.8) | 6(18.8) | 19 (59.4) | 7(21.9) | 29(23.2) | 70 (56.0) | 26(20.8) |
| Retention % | 26 (27.4) | 51 (53.7) | 18(18.9) | 7(21.9) | 16 (50.0) | 9(28.1) | 31(24.8) | 65 (52.0) | 29(23.2) |
| Recognition Discrimination Index | 15 (15.8) | 58 (61.1) | 22(23.1) | 7(21.9) | 16 (50.0) | 9(28.1) | 22(17.3) | 75 (59.1) | 30(23.7) |
| **Wechsler Memory Scale (III-A)**  **Scaled scores** |  |  |  |  |  |  |  |  |  |
| Logical Memory I | 0 (0) | 96 (100) | 0 (0) | 0(0) | 33 (100) | 0(0) | 0(0) | 127 (100) | 0(0) |
| Logical Memory II | 0 (0) | 96 (100) | 0 (0) | 0(0) | 33 (100) | 0(0) | 0(0) | 127 (100) | 0(0) |
| Family Pictures I | 0 (0) | 96 (100) | 0 (0) | 0(0) | 32 (100) | 0(0) | 0(0) | 127 (100) | 0(0) |
| Family Pictures II | 0 (0) | 92 (100) | 0 (0) | 0(0) | 32 (100) | 0(0) | 0(0) | 125 (100) | 0(0) |
| **Hamilton Depression T-Score** |  |  |  |  |  |  |  |  |  |
| T-Scores | 38 (43.2) | 44 (50.0) | 6(6.9) | 9(30) | 16 (53.3) | 5(16.7) | 46(38.6) | 62 (52.1) | 11(9.3) |
