# Supplementary material for: Neuropsychological outcomes from constant current deep brain stimulation for Parkinson's disease
Source: Mov Disord. 2016 Oct 18;32(3):433–40. doi: 10.1002/mds.26827 (PMC5363377; doi:10.1002/mds.26827)
Supplement: Supplementary file 4 — Supplementary Information Table 3. [file MDS-32-433-s004.docx]

Table 3 Supplemental: Correlations between Change in Letter, Category and Switching Fluency at 3 months and Baseline Age, Levodopa Equivalent Dose, and Attention Scores

|  | **L-Dopa Equivalent** | **Age** | **Group** | **SNST Interference** | **Trails B** |
| --- | --- | --- | --- | --- | --- |
| **Letter Fluency** | -0.05709 | -0.07185 | 0.10963 | -0.02027 | 0.00413 |
| p-value | 0.5288 | 0.4278 | 0.2255 | 0.8239 | 0.9646 |
| N | 124 | 124 | 124 | 123 | 118 |
|  |  |  |  |  |  |
| **Category Fluency** | -0.05236 | -0.0091 | 0.09128 | -0.01092 | 0.16056 |
| p-value | 0.5635 | 0.9201 | 0.3133 | 0.9046 | 0.0824 |
| N | 124 | 124 | 124 | 123 | 118 |
|  |  |  |  |  |  |
| **Switching** | -0.09064 | 0.08045 | 0.072 | 0.05934 | -0.00350 |
| p-value | 0.3168 | 0.3744 | 0.4246 | 0.5144 | 0.9707 |
| N | 124 | 124 | 124 | 123 | 113 |
