# Supplementary material for: Neuropsychological outcomes from constant current deep brain stimulation for Parkinson's disease
Source: Mov Disord. 2016 Oct 18;32(3):433–40. doi: 10.1002/mds.26827 (PMC5363377; doi:10.1002/mds.26827)
Supplement: Supplementary file 5 — Supplementary Information Table 4. [file MDS-32-433-s005.docx]

Table 4 Supplemental: Mean PDQ-Communication Change between Letter Fluency Decliners/Non-Decliners, Category Fluency Decliners/Decliners, Switching Fluency Decliners/Non-Decliners at 12 months

| Letter Fluency | N | Mean | Std Dev | P-value |
| --- | --- | --- | --- | --- |
| **Non-Decliners** | 85 | -3.24 | 21.05 | 0.027 |
| **Decliners** | 39 | 5.77 | 20.34 |  |
| **Diff (1-2)** |  | -9.02 | 20.83 |  |
| Category Fluency | N | Mean | Std Dev | P-value |
| **Non-Decliners** | 80 | -0.83 | 21.49 | 0.760 |
| **Decliners** | 44 | 0.38 | 20.80 |  |
| **Diff (1-2)** |  | -1.21 | 21.24 |  |
| Switching Fluency | N | Mean | Std Dev | P-value |
| **Non-Decliners** | 71 | -2.70 | 23.56 | 0.145 |
| **Decliners** | 53 | 2.67 | 17.20 |  |
| **Diff (1-2)** |  | -5.37 | 21.09 |  |
